# Metabolomic Analysis of Breast Cancer in Colombian Patients: Exploring Molecular Signatures in Different Subtypes and Stages

**DOI:** 10.3390/ijms26157230

**Published:** 2025-07-26

**Authors:** Lizeth León-Carreño, Daniel Pardo-Rodriguez, Andrea Del Pilar Hernandez-Rodriguez, Juliana Ramírez-Prieto, Gabriela López-Molina, Ana G. Claros, Daniela Cortes-Guerra, Julian Alberto-Camargo, Wilson Rubiano-Forero, Adrian Sandoval-Hernandez, Mónica P. Cala, Alejandro Ondo-Mendez

**Affiliations:** 1 Metabolomics Core Facility—MetCore, Vice-Presidency for Research, Universidad de los Andes, Bogotá 110111, Colombia; l.leonc@uniandes.edu.co (L.L.-C.); d.pardorodriguez@uniandes.edu.co (D.P.-R.); 2 Clinical Research Group, School of Medicine and Health Sciences, Universidad del Rosario, Bogotá 110011, Colombia; andread.hernandez@urosario.edu.co (A.D.P.H.-R.); juliana.ramirezp@urosario.edu.co (J.R.-P.); gabriela.lopezm@urosario.edu.co (G.L.-M.); ana.claros@urosario.edu.co (A.G.C.); daniela.cortesg@urosario.edu.co (D.C.-G.); 3 Biochemistrry, Cancer and Radiobiology Research Incubator, School of Medicine and Health Sciences, Universidad del Rosario, Bogotá 111221, Colombia; 4 Hospital Universitario Mayor-Méderi, Bogotá 110011, Colombia; jac8019@gmail.com (J.A.-C.); wilson.rubiano@mederi.com.co (W.R.-F.); 5 Grupo de Muerte Celular, Instituto de Genética, Universidad Nacional de Colombia-Sede Bogotá, Avenida Carrera 30, No. 45-03, Bogotá 110111, Colombia; agsandovalh@unal.edu.co

**Keywords:** metabolomic analysis, breast cancer, molecular signatures, cancer subtypes, cancer stages, amino acid metabolism, energy metabolism

## Abstract

Breast cancer (BC) is a neoplasm characterized by high heterogeneity and is influenced by intrinsic molecular subtypes and clinical stage, aspects that remain underexplored in the Colombian population. This study aimed to characterize metabolic alterations associated with subtypes and disease progression in a group of newly diagnosed, treatment-naive Colombian women using an untargeted metabolomics approach. To improve metabolite coverage, samples were analyzed using LC-QTOF-MS and GC-QTOF-MS, along with amino acid profiling. The Luminal B subtype exhibited elevated levels of long-chain acylcarnitines and higher free fatty acid concentrations than the other subtypes. It also presented elevated levels of carbohydrates and essential glycolytic intermediates, suggesting that this subtype may adopt a hybrid metabolic phenotype characterized by increased glycolytic flux as well as enhanced fatty acid catabolism. Tumor, Node, and Metastasis (TNM) staging analysis revealed progressive metabolic reprogramming of BC. In advanced stages, a sustained increase in phosphatidylcholines and a decrease in lysophosphatidylcholines were observed, reflecting lipid alterations associated with key roles in tumor progression. In early stages (I-II), plasma metabolites with high discriminatory power were identified, such as glutamic acid, ribose, and glycerol, which are associated with dysfunctions in energy and carbohydrate metabolism. These results highlight metabolomics as a promising tool for the early diagnosis, clinical follow-up, and molecular characterization of BC.

## 1. Introduction

Breast cancer (BC) is the most commonly diagnosed cancer and the second leading cause of mortality among women worldwide [1]. According to the latest report by the Global Cancer Observatory GLOBOCAN, in 2022, there were 2,296,840 new cases and 666,103 deaths. In the United States, approximately 320,000 new cases of invasive BC and 42,000 deaths are expected in 2025 [2]. In Colombia, BC is the most prevalent malignancy among women and the leading cause of cancer-related mortality, with 17,018 cases and 4752 deaths in 2022 [1]. However, the interplay of genetic, environmental, and lifestyle factors drives BC development and progression, making it a highly complex and heterogeneous disease [3,4,5,6]. Therefore, diagnostic and prognostic tools lack sufficient predictive capacity across disease stages. BC is clinically and molecularly classified into [7] different subtypes and stages with different biological, molecular, and clinical outcomes According to the eighth edition of the Tumor, Node, and Metastasis (TNM) system of the American Joint Committee on Cancer (AJCC) of 2018, this disease is classified according to the anatomical information of the tumor, as well as the expression of estrogen receptors (ER), progesterone (PR), and human epidermal growth receptor-2 (HER2) [7,8]. 

The TNM system is primarily based on tumor size, lymph node spread, and distant site metastasis [9,10]. In general, the T category describes the size of the primary tumor considering the following criteria: Tx: it is not possible to evaluate the tumor; T0: there is no evidence of the tumor; Tis: ductal carcinoma in situ; T1, T2, T3, and T4: indicate the size of the tumor, the higher the number indicates a larger tumor and/or greater proliferation to the surrounding tissues [7,8]. Thus, the anatomical stage of the tumor is classified from I to IV, where stage I refers to BC in its initial stage and stage IV when it is already advanced and metastasizes [3,9].

BC is also classified into different subtypes according to the expression of cell proliferation markers, such as Ki67, and hormone receptors: ER+, PR+, HER2+, and triple-negative (TNBC), when none of the above are present [10,11]. These subtypes include Luminal A (LA), Luminal B (LB), HER2, and Basal-Like (BL) subtypes. The Luminal A subtype is characterized by positive ER or PR expression, negative HER2 expression, and Ki-67 expression <13 [11]. Clinically, this subtype is considered low-grade, tends to grow slowly, has a favorable prognosis, has a higher survival rate, and typically responds positively to hormonal therapy [11,12]. The Luminal B subtype is also positive for ER or PR but is characterized by high proliferative activity, as evidenced by the elevated Ki-67 expression (>20%), and may be either HER2-positive or HER2-negative. Compared with LA, LB tumors have a higher growth rate and a poorer prognosis [8,10,11]. The HER2 subtype exhibited HER2 overexpression in the absence of ER and PR. These tumors tend to be more aggressive, exhibit rapid growth, and have a worse prognosis than previous tumors. Finally, basal-like tumors, or TNBC, are negative for ER, PR, and HER2. It represents approximately 20% of all BC cases, being more prevalent in women under the age of 40, and is characterized by a high rate of cell proliferation and greater aggressiveness [11].

High-throughput omics technologies have transformed our understanding of cancer in recent years. In BC, transcriptome-based analysis has led to the identification of distinct molecular subtypes; for example, TNBC has been stratified into six subtypes through transcriptomic analysis, highlighting its complex heterogeneity [13,14]. Genetic molecular tools, such as Oncotype and Mammaprint, have been developed to refine subtype classification, define prognosis, and thereby guide therapeutic decisions [13]. Therefore, a thorough understanding of tumoral biology may impact the treatment course in the future [15].

Based on these molecular insights, hormone receptor status and gene expression profiles significantly impact key metabolic pathways and physiological mechanisms, particularly those involved in energy generation and biosynthesis. These alterations generate specific metabolic fingerprints that can serve as biomarkers for different disease phenotypes [10,11,16]. Because of the dynamic nature of the metabolome, metabolite levels can change under pathological conditions, reflecting underlying metabolic dysfunctions, particularly in complex diseases such as breast cancer [3,16,17,18]. In this regard, metabolomics is a promising tool for understanding the molecular heterogeneity of BC, offering insights into the metabolic alterations that occur during cancer development and disease progression [3,19,20].

Several studies have been conducted using plasma, serum, or tissue samples to discover novel biomarkers for the early diagnosis and prediction of clinical outcomes in BC [19,20,21,22,23,24,25,26,27,28]. In addition, both intrinsic and extrinsic factors contribute to the complexity of cancer development and progression. Intrinsic factors include genetic alterations and the tissue of origin, whereas extrinsic factors include nutrient and oxygen availability, tumor microenvironment interactions, and radiation or chemotherapy exposure [29]. Together, these elements shape the tumor landscape, highlighting the importance of population-specific variables, such as genetic background, ethnicity, dietary habits, and lifestyle, in determining cancer susceptibility, progression dynamics, recurrence risk, and mortality rates for certain cancer types [29,30,31].

Although metabolomics has advanced significantly, a comprehensive characterization of metabolic profiles across intrinsic biological subtypes and disease stages is lacking. In this context, this study aimed to investigate the metabolic signatures of plasma samples from previously untreated patients with BC using untargeted metabolomics. Liquid chromatography and gas chromatography coupled with high-resolution mass spectrometry were employed to achieve a detailed metabolic characterization. The findings of this research will contribute to a deeper understanding of the molecular heterogeneity of BC in Colombian patients, allowing the development of tailored therapeutic approaches and individualized diagnostics.

## 2. Results

### 2.1. Description of Clinical Variables

A cross-sectional descriptive study was conducted to perform an exploratory analysis of serum metabolomics in a cohort of women diagnosed with BC (*n* = 141) and a control group (*n* = 14). The clinical variables included age, body mass index (BMI), and comorbid conditions across various biological subtypes and tumor stages. The average age of patients with BC was 65.1 ± 11.1 years, which was significantly higher than that of the control group (51.7 ± 13.7 years, *p* < 0.0009). This finding suggests that patients with BC in this cohort were generally older than those in the control group. Other clinical variables, including type 2 diabetes mellitus (T2DM), dyslipidemia, and hormonal status, showed no statistically significant differences between the two groups (Table S1).

The initial BC cohort was classified into the four biological subtypes: Luminal A (*n* = 41), Luminal B (*n* = 69), HER2-enriched (*n* = 17), and TNB (*n* = 14). Luminal B was the most prevalent subtype, followed by Luminal A, whereas HER2-enriched and triple-negative subtypes were less represented. This distribution aligns with the expected epidemiological patterns, where hormone receptor-positive tumors (Luminal A and B) are more commonly diagnosed than HER2-enriched and TNBC subtypes [10,11]. The patients were also classified according to the TNM staging system, which assesses disease progression. The distribution across the different stages was as follows: in situ (*n* = 17), stage IA (*n* = 23), stage IIA-IIB (*n* = 64), stage IIIA-IIIC (*n* = 31), and stage IV (*n* = 6). Most patients were diagnosed at an early stage, with a smaller proportion presenting with advanced disease.

### 2.2. Untargeted Metabolomics Analysis in Breast Cancer

Untargeted metabolomic analyses were performed using liquid chromatography quadrupole time-of-flight mass spectrometry (LC-QTOF-MS), gas chromatography quadrupole time-of-flight mass spectrometry (GC-QTOF-MS), and amino acid profiling to identify discriminant metabolites among the main BC subtypes, Luminal A (LA), Luminal B (LB), basal-like (BL), and HER2, as well as across different tumor stages (I, II, III, and IV) and a healthy control group. To control for potential confounding effects, clinical variables were assessed. Following this analysis, the examination of the clusters in the Principal Component Analysis (PCA) confirmed the grouping of quality control samples within the utilized analytical platforms (Figure S1, orange dots), supporting the analytical platforms’ stability and reliability. An initial exploratory analysis was performed, including all participants regardless of comorbidities.

The PCA score plots were generated to evaluate the metabolic differences among the study groups. However, no clear separation was initially observed (Figure S2), suggesting the presence of confounding factors affecting metabolic profiles. Following a more detailed assessment of potential confounding variables, thyroid disease, specifically idiopathic primary hypothyroidism, was identified as the main factor contributing to metabolic variability in patients with BC. Because this study primarily aimed to evaluate metabolic differences across biological subtypes and disease stages, patients with this comorbidity were excluded, and a refined subset of individuals without thyroid disease was selected to reduce confounding effects. This refinement enabled a more accurate comparison, allowing metabolic alterations to be directly attributed to BC. Additionally, patients whose tumors were ultimately classified as carcinoma in situ were excluded because these lesions are not considered malignant under current oncological classification systems because of their lack of clear invasive behavior. Therefore, they do not align with the focus of this study, which is exclusively focused on invasive BC. This decision was further supported by preliminary analyses in which the presence of in situ carcinoma emerged as an important interaction variable within the multivariate model.

The final subset of participants included 63 women diagnosed with BC and 9 individuals in the control group (Table 1). BC patients had a mean age of 62.1 ± 10.9 years, compared to 54.5 ± 12.9 years in controls. BMI was comparable between the groups (25.8 ± 4.6 vs. 25.7 ± 3.1). Regarding the biological subtypes of BC, 18 patients were classified as Luminal A, 22 as Luminal B, 13 as HER2-positive, and 10 as basal-like. The mean age varies across subtypes, with Luminal A patients averaging 57.4 ± 9.1 years, Luminal B 67.5 ± 9.7 years, HER2-positive 62 ± 11.4 years, and basal-like 58.9 ± 11.8 years. Overall, no significant differences were observed in any of the analyzed variables across groups, except for age, which varied significantly among the biological subtypes, with patients with Luminal B being older than those with the other subtypes.

In terms of cancer staging (TNM classification), 54 patients had an assigned stage, whereas the remaining cases could not be precisely classified. Among the staged patients, 10 were in stage IA, 22 in stages IIA-IIB, 18 in stages IIIA-IIIC, and 4 in stage IV. The mean age for each stage was 65.05 ± 8.5 years for IA, 60.8 ± 10.4 years for IIA-IIB, 65.2 ± 12.2 years for IIIA-IIIC, and 76.2 ± 4.2 years for stage IV. No significant differences were found in the variables analyzed across tumor stages.

### 2.3. Subtype-Specific Metabolomic Signatures in BC

**Figure 1:**
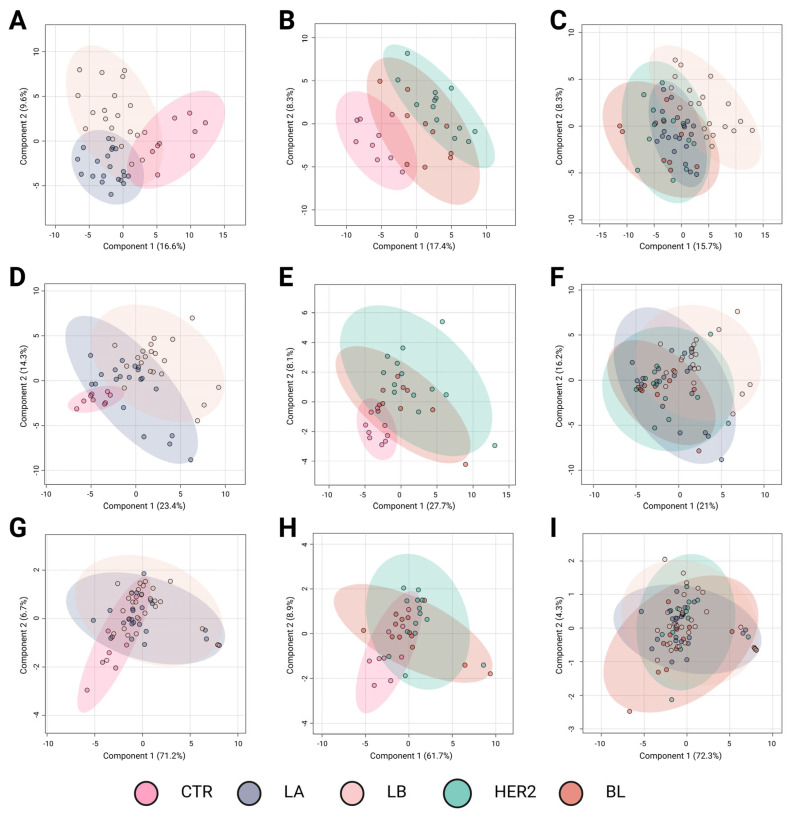
Partial Least Squares Discriminant Analysis (PLS-DA) of breast cancer subtypes based on metabolomic profiles. Panels A–C correspond to analyses performed using the LC-QTOF-MS platform, panels D–F to the GC-QTOF-MS platform, and panels G–I to amino acid profiling. (**A**) R^2^: 0.71; Q^2^: 0.42; Pval: 0.01; (**B**) R^2^: 0.91; Q^2^: 0.51; Pval: 0.01; (**C**) R^2^: 0.56; Q^2^: 0.28; Pval: 0.04; (**D**) R^2^: 0.70; Q^2^: 0.55; Pval: 0.01; (**E**) R^2^: 0.70; Q^2^: 0.11; Pval: 0.09; (**F**) R^2^: 0.35; Q^2^: 0.024; Pval: 0.02; (**G**) R^2^: 0.28; Q^2^: 0.07; Pval: 0.01; (**H**) R^2^: 0.36; Q^2^: 0.09; Pval: 0.77; and (**I**) R^2^: 0.11; Q^2^: −0.03; Pval: 0.69. Breast cancer subtypes are depicted with confidence ellipses: CTR (control, pink), LA (Luminal A, blue-gray), LB (Luminal B, beige), HER2 (Human Epidermal Growth Factor Receptor 2 (HER2-enriched, teal)), and BL (basal-like, red). The percentages on the axes indicate the variance explained by each component. LC-QTOF-MS: liquid chromatography quadrupole time-of-flight mass spectrometry, GC-QTOF-MS: gas chromatography quadrupole time-of-flight mass spectrometry.

Metabolomic profiling plays a crucial role in identifying molecular differences among BC subtypes, offering potential insights for improved classification and targeted therapy development. The results of the Partial Least Squares Discriminant Analysis (PLS-DA) revealed distinct metabolomic profiles among BC subtypes across different analytical platforms. Figure 1 presents three sets of comparative analyses to explore metabolomic differences among BC subtypes. Figure 1A,D,G compare controls (CTR) with subtypes (Luminal A and Luminal B) associated with a favorable prognosis. Figure 1B,E,H contrast controls with subtypes associated with a poorer prognosis (HER2-enriched and basal-like). Finally, Figure 1C,F,I examine the metabolic differences among the BC subtypes. Figure 1A–C correspond to LC-QTOF-MS analyses, Figure 1D–F to GC-QTOF-MS analyses, and Figure 1G–I to amino acid profiling.

The comparison between the controls and luminal subtypes with a favorable prognosis (Figure 1A,D,G) revealed a clear metabolic distinction using the LC-QTOF-MS and GC-QTOF-MS platforms. In these analyses, Figure 1A,D exhibit high R^2^ values (0.71 and 0.70, respectively) and moderate predictive power (Q^2^ = 0.42 and 0.55), indicating significant metabolic alterations in these subtypes compared with controls. In contrast, amino acid profiling (Figure 1G) showed limited discrimination ability (Q^2^ = 0.07, *p* = 0.01), suggesting that variations in amino acid metabolism do not play a key role in distinguishing these subtypes from controls.

Metabolic divergence was observed when comparing controls with the more aggressive BC subtypes (Figure 1B,E,H). The most pronounced separation occurred in the LC-QTOF-MS analysis (Figure 1B), which showed the highest R^2^ value (0.91) and considerable predictive power (Q^2^ = 0.51). Similarly, GC-QTOF-MS analysis (Figure 1E) indicated a clear group differentiation (R^2^ = 0.70), although with lower predictive power (Q^2^ = 0.11). These findings suggest significant metabolic reprogramming in the aggressive subtypes (HER2-enriched and basal-like). In contrast, amino acid profiling (Figure 1H) did not show significant separation (Q^2^ = 0.09), reinforcing the idea that amino acid metabolism alone is insufficient to differentiate these subtypes from controls.

Direct comparison among the BC subtypes (Figure 1C,F,I) revealed moderate discrimination. The separation was partial in the LC-QTOF-MS analysis (Figure 1C), with an R^2^ of 0.56 and a Q^2^ of 0.28, indicating the presence of metabolic differences among subtypes, albeit with some degree of overlap. Notably, panel C suggests a trend in metabolic profiles transitioning from subtypes with favorable prognoses to those associated with poorer outcomes. Although this trend is evident, further studies are needed to precisely define the metabolic shifts underlying this transition. In the GC-QTOF-MS analysis (Figure 1F), the discriminative ability was more limited (Q^2^ = 0.024), suggesting that some metabolic profiles were shared among the different subtypes. Finally, amino acid profiling (Figure 1I) showed the lowest predictive power (Q^2^ = −0.03), indicating that variations in amino acid metabolism did not significantly contribute to subtype differentiation.

Overall, the results indicate that the LC-QTOF-MS and GC-QTOF-MS platforms allow for better differentiation among BC subtypes, particularly between the controls and aggressive subtypes (HER2, BL). The observed separation suggests significant metabolic reprogramming in aggressive tumors, whereas luminal subtypes exhibit distinct metabolic signatures, albeit with a lower degree of differentiation. In contrast, amino acid profiling shows a limited capacity to discriminate between subtypes, suggesting that a broader metabolomic approach is necessary for a more precise classification. These findings highlight the value of metabolomic analysis in characterizing BC subtypes and their potential applications in molecular subtyping strategies and personalized therapeutic approaches.

Metabolomic analysis identified 96 altered compounds across the different BC subtypes (Supplementary Tables S2 and S3). Among these, glycerophosphocholines accounted for the highest proportion of altered metabolites (23.96%), followed by carnitines (17.71%) and amino acids (11.46%). Carbohydrates (8.33%) and fatty acids (9.37%) also contributed significantly to the metabolic differentiation among the subtypes. Less abundant, but still relevant, were alterations in other chemical families, including phosphosphingolipids (4.16%), carboxylic acids (4.16%), and bile acids (2.08%). Additional classes with a lower number of altered metabolites included steroids, beta-hydroxylated acids, ketones, and alcohols (1.04% each).

**Figure 2:**
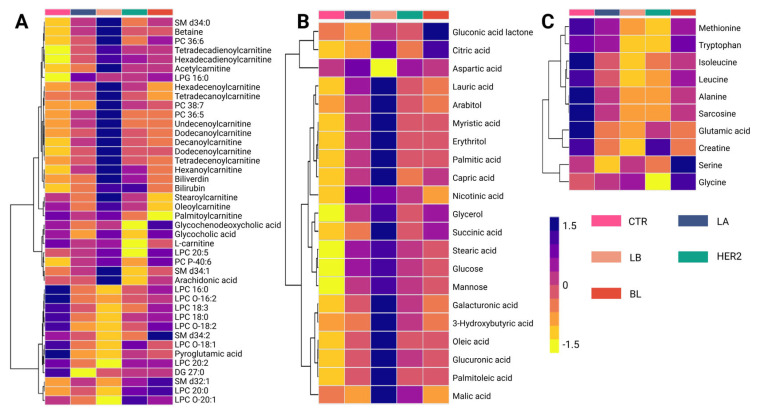
Hierarchical clustering heatmap of metabolite variations among breast cancer subtypes. (**A**) Altered metabolites identified using the LC-QTOF-MS platform. (**B**) Altered metabolites identified using the GC-QTOF-MS platform. (**C**) Altered amino acid profiles. The heatmap color scale indicates variations in metabolite levels, where purple and yellow represent increased and decreased metabolites, respectively. The subtypes are presented according to the figure legend. LA (Luminal A, blue-gray), LB (Luminal B, beige), HER2 (Human Epidermal Growth Factor Receptor 2 (HER2-enriched, teal)), and BL (basal-like, red), SM: Sphingomyelin, PC: Phosphatidylcholine, LPG: Lysophosphatidylglycerol, LPC: Lysophosphatidylcholine, DG: Diacylglycerol, CTR: Control, LB: Luminal B, BL: Basal-like, LC-QTOF-MS: liquid chromatography quadrupole time-of-flight mass spectrometry, GC-QTOF-MS: gas chromatography quadrupole time-of-flight mass spectrometry.

A detailed analysis of the altered metabolites among BC subtypes is presented in the hierarchical clustering heatmap, where purple indicates increased metabolite levels and yellow indicates decreased levels (Figure 2). The metabolic changes observed using the LC-QTOF-MS platform (Figure 2A) revealed three distinct clusters. The first cluster consisted mainly of carnitines, which showed reduced levels in the control group and increased levels in cancer subtypes, with the Luminal B subtype exhibiting the highest levels of these metabolites. Conversely, the third cluster was primarily composed of lysophospholipids, which increased in the control group but decreased in the initial subtypes, Luminal A and Luminal B. Finally, the intermediate cluster, which is heterogeneous and composed mainly of fatty acids, did not exhibit a consistent trend across comparisons, suggesting variability in lipid metabolism alterations among BC subtypes. This distinct metabolic clustering highlights subtype-specific alterations, suggesting a potential role of carnitines and lysophospholipids in metabolic reprogramming associated with BC progression.

In contrast, the analysis of metabolic variations observed using the GC-QTOF-MS platform (Figure 2B) revealed distinct clustering patterns among BC subtypes. Three main clusters were identified in this study. The first cluster was composed mainly of organic acids, including citric acid, aspartic acid, and gluconic acid lactone. These metabolites show decreased levels in the control group and increased levels in BC subtypes, particularly in the basal-like (BL) subtype. Similarly, the second and third clusters consisted primarily of fatty acids and carbohydrates, such as palmitic acid, oleic acid, capric acid, glucose, succinic acid, and glycerol, which were reduced in the control group but showed an increasing trend across all subtypes. Notably, the most pronounced metabolic changes were observed in the Luminal B subtype, suggesting a more extensive metabolic reprogramming in this group. This pattern may reflect significant disruptions in lipid metabolism and other energy-production pathways associated with cancer progression.

Analysis of amino acid levels across BC subtypes (Figure 2C) revealed distinct clustering patterns. The first cluster is composed mainly of essential and branched-chain amino acids, including methionine, tryptophan, leucine, and isoleucine, which show lower levels in cancer subtypes compared to the control group. Similarly, alanine, sarcosine, glutamic acid, and creatinine levels also exhibited a decreasing trend in cancer subtypes. Conversely, serine and glycine levels were higher in the BL subtype than in the controls. Overall, these findings highlight metabolic reprogramming, particularly amino acid utilization, as a key feature of BC progression.

### 2.4. Stage-Specific TNM Metabolomic Signatures in BC

**Figure 3:**
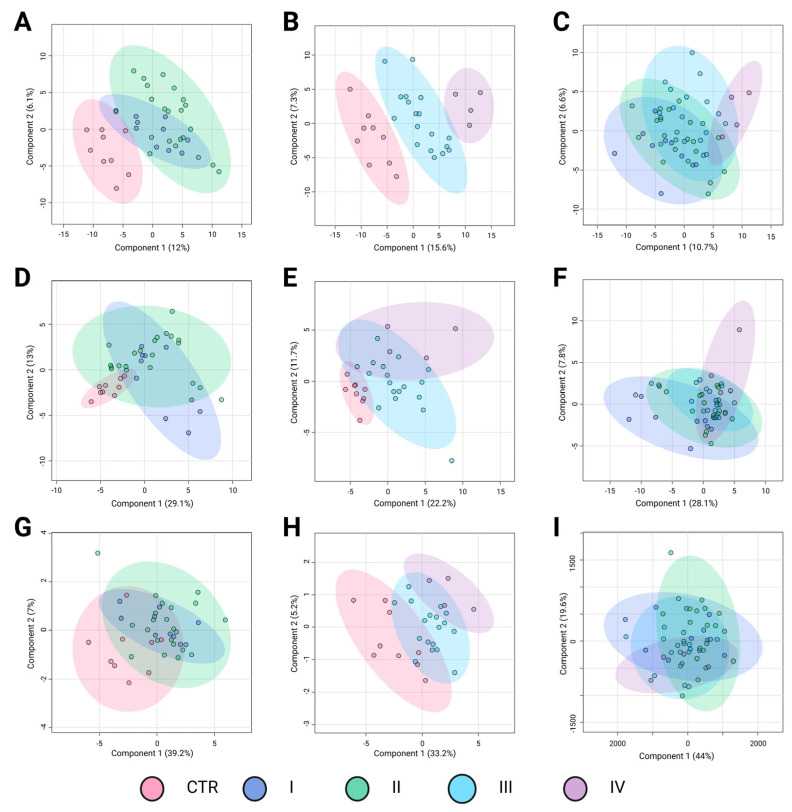
Partial Least Squares Discriminant Analysis (PLS-DA) of breast cancer stages based on metabolomic profiles. Panels A–C correspond to analyses performed using the LC-QTOF-MS platform, panels D–F to the GC-QTOF-MS platform, and panels G–I to amino acid profiling. (**A**) R^2^: 0.52; Q^2^: 0.22; Pval: 0.01; (**B**) R^2^: 0.91; Q^2^: 0.62; Pval: 0.03; (**C**) R^2^: 0.32; Q^2^: 0.01; Pval: 0.32; (**D**) R^2^: 0.19; Q^2^: −0.17; Pval: 0.11; (**E**) R^2^: 0.72; Q^2^: 0.31; Pval: 0.19; (**F**) R^2^: 0.49; Q^2^: 0.02; Pval: 0.31; (**G**) R^2^: 0.23; Q^2^: 0.07; Pval: 0.19; (**H**) R^2^: 0.50; Q^2^: 0.35; Pval: 0.004; and (**I**) R^2^: 0.12; Q^2^: −0.02; Pval: 0.51. Breast cancer subtypes are depicted using confidence ellipses: Control group (CTR (pink)), Stage I (blue), Satge II (green), Stage III (blue sky), and Stage IV (purple). The percentages on the axes indicate the variance explained by each component. LC-QTOF-MS: liquid chromatography quadrupole time-of-flight mass spectrometry, GC-QTOF-MS: gas chromatography quadrupole time-of-flight mass spectrometry.

Similar to BC subtypes, stratification by TNM stage may correlate with distinct metabolomic alterations, reflecting the progressive metabolic reprogramming associated with tumor advancement. The PLS-DA results in Figure 3 illustrate the metabolomic differentiation across BC stages based on three key comparisons: control (CTR) versus early-stage cancer (I and II), control versus advanced-stage cancer (III and IV), and direct comparisons among cancer stages. The different groups are represented by confidence ellipses in distinct colors: CTR is shown in pink, early-stage cancer stages I and II are represented in blue and green, respectively, whereas advanced-stage cancer stages III and IV are depicted in light blue and purple, respectively.

The first comparison, control versus early-stage cancer (Figure 3A,D,G), showed moderate separation depending on the analytical platform. In the LC-QTOF-MS analysis (Figure 3A), the clustering suggested some metabolic differentiation (R^2^ = 0.52, Q^2^ = 0.22), although with noticeable overlap, particularly between CTR and stage I. The GC-QTOF-MS analysis (Figure 3D) revealed a weaker separation (R^2^ = 0.19, Q^2^ = −0.17), indicating substantial metabolic similarity between the control and early-stage samples in this platform. Similarly, amino acid profiling (Figure 3G) shows limited discrimination (R^2^ = 0.23, Q^2^ = 0.07), suggesting that amino acid metabolism alone does not strongly differentiate early-stage BC from healthy controls.

The second comparison, control versus advanced-stage cancer (Figure 3B,E,H) demonstrated a more pronounced metabolic shift. In the LC-QTOF-MS analysis (Figure 3B), there was a strong separation (R^2^ = 0.91, Q^2^ = 0.62), suggesting significant metabolic alterations in the advanced stages compared to the controls. The GC-QTOF-MS platform (Figure 3E) also showed moderate discrimination (R^2^ = 0.72, Q^2^ = 0.31), although there was some degree of overlap. In contrast, amino acid profiling (Figure 3H) reveals a clearer distinction (R^2^ = 0.50, Q^2^ = 0.35), indicating that amino acid metabolism may contribute more prominently to metabolic changes in later stages of the disease.The direct comparison among cancer stages (Figure 3C,F,I) revealed more variable results. In LC-QTOF-MS (Figure 3C), the metabolic profiles exhibited some degree of transition between the early and advanced stages, though with overlapping clusters (R^2^ = 0.32, Q^2^ = 0.01). GC-QTOF-MS (Figure 3F) showed slightly better separation (R^2^ = 0.49, Q^2^ = 0.02), although the predictive power remained low. Amino acid profiling (Panel I) demonstrated the weakest discrimination (R^2^ = 0.12, Q^2^ = −0.02), suggesting that amino acid metabolism alone does not provide clear stratification among cancer stages.

Overall, these results suggest that metabolic alterations are more pronounced in advanced cancer stages, with LC-QTOF-MS showing the strongest separation between the early and advanced stages. However, the overlap in intra-stage comparisons highlights the need for further refinement of metabolomic markers to improve BC stage classification.

**Figure 4:**
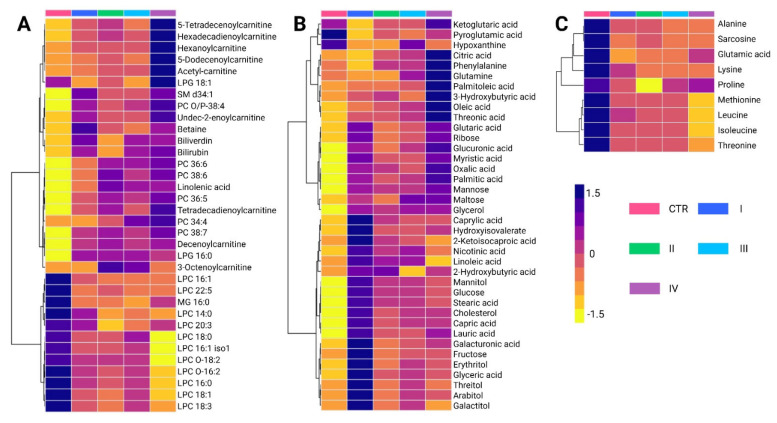
Hierarchical clustering heatmap of metabolite variations among breast cancer stages. (**A**) Altered metabolites identified using the LC-QTOF-MS platform. (**B**) Altered metabolites identified using the GC-QTOF-MS platform. (**C**) Altered amino acid profiles. The heatmap color scale indicates variations in metabolite levels, where purple and yellow represent increased and decreased metabolites, respectively. The stages (I, II, III and IV) and Control (CTR) are presented according to the figure legend. PC: Phosphatidylcholine, LPC: Lysophosphatidylcholine, LPG: Lysophosphatidylglycerol, MG: Monoacylglycerol SM: Sphingomyelin, LC-QTOF-MS: liquid chromatography quadrupole time-of-flight mass spectrometry, GC-QTOF-MS: gas chromatography quadrupole time-of-flight mass spectrometry.

A total of 84 metabolites exhibited significant alterations across different BC stages (Supplementary Tables S4 and S5). Among the predominant chemical families, glycerophosphocholines (20.2%) and carbohydrates (17.8%) were the most abundant, followed by fatty acids (13.1%) and carnitines (10.7%). Amino acids accounted for 10.7% of the altered metabolites, whereas bilirubin, carboxylic acids, hydroxy acids, and phosphosphingolipids contributed less than 5%. Metabolomic profiling using the LC-QTOF-MS platform (Figure 4A) revealed distinct clustering patterns across BC stages (I–IV). Two principal clusters were identified. The first cluster consisted primarily of glycerophospholipids and carnitines, which showed increased levels in cancer stages compared to controls, with stage IV exhibiting the highest levels of these metabolites. Conversely, the second cluster was mainly composed of lysophosphatidylcholines, showing a progressive decline across cancer stages relative to the control group.

Analysis of metabolite variations using the GC-QTOF-MS platform (Figure 4B) revealed significant alterations in fatty acids, carbohydrates, and certain amino acids across BC stages. Overall, these metabolites were elevated in cancer stages compared to healthy controls, reflecting metabolic reprogramming associated with disease progression. Notably, the first cluster primarily consists of fatty acids, such as palmitic acid and oleic acid, along with amino acids, including phenylalanine and glutamine. These metabolites also showed a progressive increase across cancer stages, with the most pronounced elevations observed in stage IV.

The intermediate cluster was mainly composed of organic acids and sugar alcohols, including ribose, mannose, and maltose. These metabolites are generally elevated in cancer stages compared to healthy controls, with some reaching particularly high levels in stages II and IV. The lower cluster includes metabolites such as cholesterol, linoleic acid, glucose, and stearic acid, along with capric acid, lauric acid, and galacturonic acid. Interestingly, these metabolites were most abundant in stage I cancer, and their levels decreased in more advanced cancer stages. This pattern suggests that early metabolic disruptions evolve as the disease progresses. Finally, Figure 4C reveals similar trends in the behavior of certain amino acids, such as alanine, sarcosine, lysine, and leucine, which were found at higher levels in healthy controls compared to cancer stages. This pattern is consistent with the observations made in the analysis of biological subtypes.

### 2.5. Identification of Potential Diagnostic Biomarkers for BC Patients

**Figure 5:**
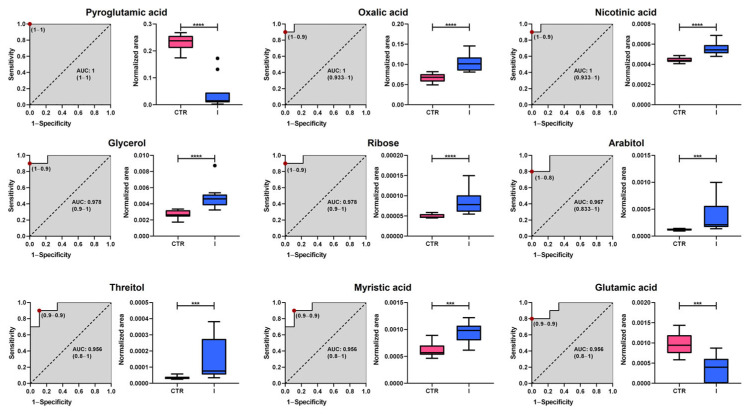
Analysis of Receiver Operating Characteristic curves of potential plasma biomarkers for distinguishing between healthy controls and women with stage I breast cancer. Box plots in pink and blue represent the control (CTR) and stage I breast cancer groups, respectively. Data are presented as median ± standard deviation. Asterisks indicate the level of statistical significance for differences between groups: *** *p* < 0.001, **** *p* < 0.0001. AUC: Area Under the Curve.

**Figure 6:**
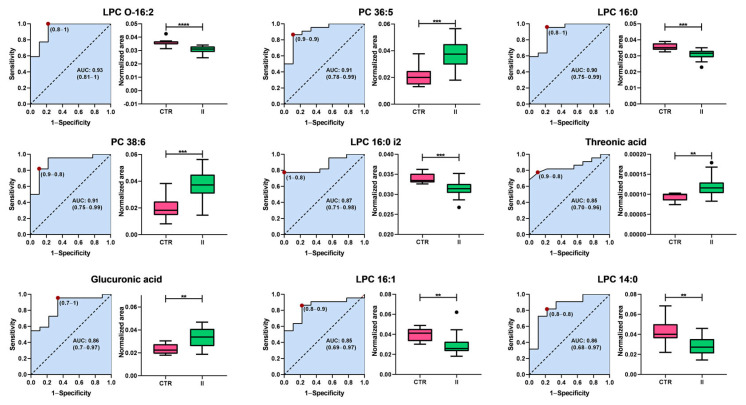
Analysis of the Receiver Operating Characteristic curves of potential plasma biomarkers for distinguishing between healthy controls and women with stage II breast cancer. Box plots in pink and green represent the control (CTR) and stage II breast cancer groups, respectively. Data are presented as median ± standard deviation. Asterisks indicate the level of statistical significance for differences between groups: ** *p* < 0.01, *** *p* < 0.001, **** *p* < 0.0001. AUC: Area Under the Curve.

To identify potential early-stage (stages I and II) BC biomarkers, a Receiver Operating Characteristic (ROC) curve analysis was performed on a set of statistically significant plasma metabolites, which were selected following false discovery rate (FDR) correction and achieving a level 2 identification. Figure 5 and Figure 6 show the nine metabolites with the highest discriminatory capacity between healthy controls and patients with stage I or II BC.

The analysis revealed substantial diagnostic potential, with Area Under the Curve (AUC) values ranging from 0.867 to 1.000. Among the most promising candidates, pyroglutamic acid, nicotinic acid, and oxalic acid exhibit perfect classification performance (AUC = 1.000), highlighting their potential as highly specific biomarkers. Additionally, glycerol, ribose, and myristic acid demonstrated excellent predictive capacity, with AUC values above 0.95, while threitol and glutamic acid also showed robust discrimination (AUC = 0.956 and 0.896, respectively). Although arabitol had a slightly lower performance (AUC = 0.867), it still exhibited notable classification ability. Box plots further illustrate the significant differences in metabolite abundance between groups, with healthy controls represented in pink and stage I BC patients in blue. These findings suggest that the identified metabolites could serve as promising early-stage BC biomarkers, warranting further validation in larger independent cohorts.

Interestingly, the metabolic profile distinguishing healthy controls from patients with stage II BC contrasts sharply with that observed in stage I. While the primary biomarkers differentiating stage I BC are associated with energy metabolism, particularly carbohydrates, the classification between controls and stage II patients is predominantly driven by alterations in glycerophospholipid metabolism. The Area Under the Curve (AUC) values range from 0.80 to 0.91, demonstrating a strong classification capacity. Among these, lysophosphatidylcholines (LPCs) and phosphatidylcholines (PCs) represent the most prominent classes of biomarkers. This shift suggests metabolic reprogramming during disease progression, in which lipid dysregulation emerges as a key feature in the later stages of BC.

Lysophosphatidylcholines, including LPC O-16:2 (AUC = 0.83), LPC 16:0 (AUC = 0.86), LPC 16:0 i2 (AUC = 0.87), LPC 16:1 (AUC = 0.85), and LPC 14:0 (AUC = 0.80), exhibited lower levels in patients with stage II BC than in controls, suggesting a disruption in phospholipid metabolism. In contrast, PC 36:5 (AUC = 0.91) and PC 38:6 (AUC = 0.87) levels increased in stage II patients. In addition to the lipid metabolites, two non-lipid compounds, threonic acid (AUC = 0.85) and glucuronic acid (AUC = 0.86), were identified as potential biomarkers. Both metabolites exhibited increased levels in patients with stage II BC compared with controls, suggesting additional metabolic alterations beyond lipid dysregulation.

These findings highlight the shift in metabolic signatures associated with the progression of BC. In early stages (stage I), carbohydrate metabolism plays a central role, with key metabolites involved in energy production emerging as the most relevant biomarkers. However, as the disease advances to stage II, glycerophospholipid metabolism becomes the dominant feature, with a significant decrease in LPCs and an increase in PCs, suggesting profound alterations in lipid remodeling pathways. Additionally, the observed increase in non-lipid metabolites, such as threonic acid and glucuronic acid, further supports the notion of metabolic reprogramming during tumor progression. These metabolic shifts warrant further investigation of their mechanistic roles and potential utility as biomarkers for early detection and disease monitoring.

## 3. Discussion

This study provides novel insights into the metabolic heterogeneity of BC by examining the effects of intrinsic subtypes and disease stages on the metabolic profiles. Metabolic heterogeneity has been associated with various factors, including genetic variations, environmental influences, and tumor microenvironment [5,6,10,11,31,32,33,34]. While such diversity has been documented in other populations, it remains underexplored in Colombian women [30,35,36,37]. Using untargeted metabolomics, distinct metabolic signatures have been identified across different subtypes and disease stages in women aged 25–80 years, newly diagnosed with BC, and without prior systemic treatment. The findings align with some previously reported results while also showing discrepancies with others. These results enhance our understanding of the molecular diversity of BC and suggest potential biomarkers for subtype classification and prognosis, with particular relevance to this population.

As an initial approach, this study assessed the changes in metabolic profiles associated with the intrinsic biological subtypes of BC (Luminal A, Luminal B, HER2-enriched, and TNBC). Distinct metabolic patterns emerged for each subtype, particularly when subtypes were grouped according to prognosis and compared to healthy controls. While certain metabolic signatures were shared among subtypes, notable differences were observed, particularly between those associated with favorable prognoses (e.g., Luminal A) and more aggressive phenotypes (e.g., TNBC). A gradual shift in metabolic profiles along this prognostic continuum was also evident, suggesting a possible metabolic reprogramming progression. This trend is consistent with previous observations indicating that BC subtypes may exhibit a degree of phenotypic plasticity, wherein tumors could potentially evolve from one subtype to another over time or in response to therapeutic pressure [38,39,40,41]. Such dynamics suggest that subtypes may not be entirely fixed categories, highlighting the relevance of metabolomic profiling for gaining insights into tumor evolution and potentially guiding prognosis and personalized treatment approaches.

Further multivariate analysis using PLS-DA confirmed the discriminative power of untargeted metabolomic approaches, with LC-QTOF-MS and GC-QTOF-MS outperforming amino acid profiling, particularly in distinguishing healthy controls from aggressive subtypes, such as HER2-enriched and TNBC. The observed dysregulation of key metabolite classes, including phosphatidylcholines, carbohydrates, carnitines, fatty acids, and amino acids, is consistent with previous reports [21,22,24,26,28,36,42,43,44,45,46,47,48,49,50]. These metabolic alterations likely reflect the underlying biological heterogeneity and adaptive capabilities of more aggressive subtypes. Notably, the capacity of these tumors to reprogram their metabolism to utilize a broad array of carbon sources, including glucose, amino acids, and lipids, appears to be a crucial feature supporting their survival and expansion in nutrient-variable microenvironments. This metabolic flexibility is not only a hallmark of malignancy but also a key driver of disease progression. Collectively, our results are consistent with those of previous studies reporting intensified metabolic reprogramming in BC with poor prognosis [22,36,45,51,52].

Although many observed alterations were consistent across subtypes, the Luminal B subtype stood out because of its more pronounced patterns. Specifically, Luminal B tumors exhibited significantly higher levels of long-chain acylcarnitines, such as palmitoylcarnitine, stearoylcarnitine, and oleoylcarnitine, along with increased concentrations of free fatty acids, compared to other subtypes. This suggests a preferential reliance on fatty acid oxidation for energy production, consistent with previous reports describing enhanced beta-oxidation activity in Luminal B tumors [51,53]. Moreover, the accumulation of monoacylglycerols, particularly 1-monoacylglycerols, has been linked to increased monoacylglycerol lipase activity and is associated with invasive, mesenchymal-like features in tumor cells [51,53]. These findings underscore the critical involvement of lipid metabolism in the progression of Luminal B tumors, suggesting that its dysregulation may contribute to the aggressive clinical behavior of this subtype. The pronounced activation of the lipid metabolic pathways observed in our study may be associated with the acquisition of mesenchymal features, as previously reported by Zhang et al. [54]. In that study, the authors demonstrated that overexpression of fatty acid synthase (FASN), a key enzyme involved in de novo fatty acid synthesis, promotes epithelial-to-mesenchymal transition through post-translational modifications of essential regulatory proteins [55,56,57]. This mechanism may explain the elevated fatty acid levels detected in Luminal B tumors and their potential role in promoting tumor progression and phenotypic plasticity.

Alterations in carbohydrate metabolism have also emerged as key features that distinguish patients with BC from healthy individuals. Elevated levels of carbohydrates and essential glycolytic intermediates, including glucose 6-phosphate, fructose 6-phosphate, and fructose 1,6-bisphosphate, have been previously reported in the context of the Warburg effect, which describes a metabolic shift toward aerobic glycolysis that is commonly observed in rapidly proliferating tumor cells. These metabolic changes were also observed in this study. Notably, our study revealed that the highest concentrations of carbohydrates were observed in Luminal B tumors, rather than in the HER2-enriched or TNBC subtypes, which are typically associated with greater aggressiveness and higher glycolytic activity [3,46,51,58]. This interesting finding may reflect underlying metabolic heterogeneity within the Luminal B group and suggests that this subtype may adopt a hybrid metabolic phenotype characterized by increased glycolytic flux as well as enhanced fatty acid catabolism [59,60,61].

In addition, amino acid levels were consistently lower in patients with BC than in healthy controls. This pattern may reflect increased metabolic demands associated with biosynthetic processes, redox homeostasis, or anaplerotic flux, as previously reported [53]. Several studies have suggested that this reduction may result from the preferential utilization of amino acids to support rapid tumor cell proliferation [42,49,62]. For instance, Huang et al. (2023) identified the alanine, aspartate, and glutamate pathways as particularly relevant for early BC detection, noting a marked decrease in the abundance of metabolites involved in these pathways in patients compared to healthy individuals [62]. Taken together, these results highlight the complexity of metabolic reprogramming in BC and suggest the existence of subtype-specific metabolic adaptations that support tumor growth and progression.

While molecular subtypes are key determinants of BC biology, the clinical stage also plays a critical role in shaping the tumor’s metabolic landscape [63,64]. Beyond the subtype-specific metabolic adaptations discussed above, our metabolomic analysis revealed distinct metabolic signatures associated with disease progression. These stage-dependent alterations reflect the evolving metabolic demands of tumors as they advance and may be indicative of increased proliferation, invasion, and systemic interaction with the host. Therefore, exploring how metabolic profiles vary across different stages provides additional insights into tumor biology and may aid in identifying potential biomarkers for disease monitoring or therapeutic targeting.

TNM stage-stratified analysis revealed that metabolic alterations progressively evolve as BC advances. In early-stage tumors (I–II), our findings are consistent with previous reports by Hadi et al. [22] and Matos Do Canto et al. [65], who noted similar challenges in distinguishing early-stage disease based solely on metabolic profiles. However, as the disease progressed, clearer metabolic patterns began to emerge [66]. The hierarchical clustering patterns observed in both LC-QTOF-MS and GC-QTOF-MS platforms reflect stage-dependent metabolic rewiring closely associated with cellular signaling dynamics in tumor progression. The progressive increase in glycerophospholipids and carnitines identified by LC-QTOF-MS may indicate enhanced membrane biosynthesis and mitochondrial fatty acid transport, both essential processes for rapidly proliferating cancer cells. These alterations are regulated in part by oncogenic signaling pathways such as PI3K/AKT and mTOR, which promote lipid biosynthesis and energy production in response to proliferative stimuli [57,67]. In advanced disease stages (III–IV), we observed a marked increase in phosphatidylcholines and a concomitant decrease in lysophosphatidylcholines, which are consistent with and expand upon the findings reported by Jobard et al. [68]. The accumulation of phosphatidylcholines in later stages may be attributed to their dual roles as signaling molecules and essential components for membrane biosynthesis, energy storage, and key processes involved in tumor progression, such as proliferation, migration, invasion, and metastasis [69,70,71]. This metabolic shift is particularly relevant, as several pharmacological strategies targeting phospholipid metabolism are currently under investigation in preclinical BC models, potentially offering new avenues for therapeutic intervention [72,73,74]. Importantly, not all phospholipid species are associated with tumor progression. Lysophosphatidylcholine, for example, has been inversely correlated with BC risk [75], a finding supported by our observation of elevated levels in healthy controls. Similarly, Kühn et al. [76] reported that higher circulating levels of lysophosphatidylcholine were associated with a reduced risk of various cancers, including BC. This trend was also evident in our analyses stratified by both stage and molecular subtype, thus reinforcing the potential protective role of this lipid class in BC pathophysiology.

The GC-QTOF-MS dendrogram further reveals early enrichment in fatty acids and carbohydrates—including lauric acid, glucose, and linoleic acid—in stage I tumors, suggesting metabolic priming for rapid biomass generation. This is consistent with early activation of glycolytic and lipogenic programs downstream of hypoxia-inducible factors (HIFs) and MYC signaling, which enhance glucose uptake, glycolysis, and fatty acid synthesis in pre-invasive lesions [77]. The intermediate accumulation of sugar alcohols and organic acids (e.g., ribose, mannose) in stages II and IV reflects fluctuating demands in nucleotide biosynthesis and redox regulation, likely driven by dynamic engagement of the pentose phosphate pathway and oxidative stress responses [78]. The late-stage increase in proline and phenylalanine also supports metabolic adaptations favoring invasion and metastasis; proline metabolism, in particular, has been shown to regulate matrix remodeling, EMT, and ROS balance via proline dehydrogenase signaling and collagen biosynthesis [79,80]. 

Furthermore, several metabolites, such as SM d34:1, betaine, 2-undecenoylcarnitine, biliverdin, and bilirubin, displayed a nonlinear pattern across BC stages, with an increase in stage I, a decrease in stages II–III, and a new increase in stage IV. The first spike could be linked to increased cell proliferation and membrane remodeling, notably of sphingomyelins and phosphatidylcholines, which are required for tumor cell shape and signaling [26]. The decrease in intermediate stages could indicate a shift toward aerobic glycolytic metabolism, which inhibits oxidative pathways and influences betaine and acylcarnitine metabolism [81].

Conversely, amino acid profiling revealed that certain metabolites, such as alanine, sarcosine, lysine, and leucine, were more abundant in healthy controls, reflecting a trend similar to that observed in the subtype-based analysis [62,82]. These results suggest a progressive reprogramming of both lipid and amino acid metabolism as the disease progresses, potentially reflecting increased biosynthetic and energetic demands in later stages of tumor development.

Interestingly, the ability to discriminate between healthy controls and early-stage BC (stages I and II) appeared to be strongly associated with the same chemical families previously identified as metabolically altered in our profiling analyses. In stage I, key discriminating metabolites belonged to classes such as fatty acids, organic acids, and carbohydrates, whereas in stage II, the most prominent changes involved lipid classes, particularly glycerophospholipids. These observations not only reinforce the biological relevance of the identified metabolic pathways but also support their utility in clinical stratification. From a diagnostic standpoint, we identified nine plasma metabolites (including pyroglutamic acid, glycerol, and ribose) with high potential to discriminate early-stage (I) BC (AUC: 0.867–1.000), coinciding with reported alterations in energy and carbohydrate metabolism [54,55]. In stage II, the decrease in lysophosphatidylcholines (LPCs) and increase in phosphatidylcholines (PCs) reflected a transition toward lipid dysfunction [83,84], while threonic and glucuronic acids emerged as new unreported candidates. These findings, validated by recent studies [85,86,87], underscore the potential of metabolomics for developing non-invasive diagnostic panels. Future studies should evaluate their clinical applicability in larger study groups by integrating these biomarkers with early detection and tumor progression monitoring strategies.

These integrated metabolic signatures suggest that tumor cells progressively coordinate nutrient availability and metabolic pathway activation in alignment with key signaling networks to meet evolving functional demands—ranging from proliferation and immune escape to metastasis. Understanding these interactions offers potential avenues for therapeutic intervention, especially through metabolic signaling nodes such as mTOR, MYC, and HIF-1α.

Despite the robustness of our findings, this study has several limitations that should be acknowledged. First, the relatively limited sample size may restrict the generalizability of the results, particularly when patients are stratified by molecular subtype and disease stage. Expanding the sample size in future studies will be essential to improve statistical power, validate candidate biomarkers, and capture the full spectrum of metabolic variability among patients. Additionally, while we accounted for primary idiopathic hypothyroidism, other comorbidities, such as obesity, diabetes, and cardiovascular disease, were not systematically controlled and may influence metabolic profiles independently of BC. Incorporating broader clinical data and controlling for these confounding variables in future research will be critical to ensure that the observed metabolic alterations are truly cancer-specific and to better understand how comorbid conditions may contribute to BC development and progression.

## 4. Materials and Methods

### 4.1. Study Population

This cross-sectional, exploratory study with an analytical component analyzed 141 serum samples from Colombian patients who lived in Bogotá, were aged 25–80 years, and had newly diagnosed primary BC at any stage and biological subtype. Patients were recruited from May 2021 to August 2023. All patients were treated at the Hospital Universitario Mayor Méderi and consented to the collection of clinical information and blood samples for study participation. Exclusion criteria included previous adjuvant or systemic oncologic treatment, as well as a history of autoimmune diseases, genetic metabolic disorders, pharmacologic or HIV-related immunosuppression, and any other type of cancer or tumor aside from BC.

A control group of 14 samples from women without BC was also analyzed. The absence of malignancy was confirmed using mammography or breast ultrasound within the past year, with a BI-RADS score of < 2 and no medical history of any type of cancer. All control participants were alive and cancer-free at the time of sample collection. These control samples were primarily included to serve as a quality control step and help preserve the analytical stability of the models. This study was approved by the Institutional Ethics Committee of Universidad del Rosario and the Scientific Committee of the Hospital Universitario Mayor Méderi.

Participants completed a questionnaire administered in collaboration with co-investigators that collected data on the most prevalent multifactorial and metabolic comorbidities observed among Colombian women, particularly those with BC, such as dyslipidemia, primary hypothyroidism of non-autoimmune or idiopathic origin, type II diabetes mellitus, obesity, and arterial hypertension. The participants’ current height and weight were also recorded. The body mass index (BMI) was calculated as the weight in kilograms divided by the height in meters squared. The questionnaire also gathered information on age and hormonal status.

### 4.2. Assessment of Confounding Factors and Final Cohort Selection

All study participants were included as part of the initial exploratory analysis regardless of the presence of comorbidities. Score plots were generated using Principal Component Analysis (PCA) and Orthogonal Partial Least Squares Discriminant Analysis (OPLS-DA) to evaluate metabolic differences among the study groups. However, no clear separation was observed between the groups, suggesting the presence of confounding factors influencing metabolic profiles.

After a thorough evaluation of the clinical variables controlled in the study, thyroid disease, specifically idiopathic primary hypothyroidism, was identified as the main factor that significantly contributed to the metabolic variability observed in BC patients. Because the primary aim of the study was to assess metabolic differences according to the biological subtypes and clinical stages of the disease, patients with this comorbidity were excluded from further analyses.

Additionally, patients whose tumors were ultimately classified as carcinoma in situ were excluded, as these lesions are considered non-invasive neoplastic epithelial proliferation that lacks the clear invasive behavior necessary to reach a malignant stage [88] Therefore, they do not align with the focus of this study, which exclusively focuses on invasive BC. This decision was further supported by preliminary analyses in which the presence of in situ carcinoma emerged as an important interaction variable within the multivariate model.

As a result, a refined subset consisting of 63 cases of invasive BC without thyroid disease and 9 healthy controls was selected to reduce the impact of confounding factors. This cohort refinement allowed for a more accurate comparison between the groups, allowing the observed metabolic alterations to be more directly attributed to BC.

### 4.3. Untargeted Metabolomics Analysis

Untargeted metabolomics analyses were performed using liquid chromatography quadrupole time-of-flight mass spectrometry (LC-QTOF-MS), gas chromatography quadrupole time-of-flight mass spectrometry (GC-QTOF-MS), and amino acid profiling to identify discriminant metabolites among the main BC subtypes—Luminal A (LA), Luminal B (LB), basal-like (BL), and HER2-positive (HER2)—as well as among different tumor stages (I, II, III, IV) and a healthy control group. Clinical variables were assessed to control for possible confounding factors. Following this analysis, sample clustering in PCA confirmed the grouping of quality control (QC) samples within the analytical platforms used (Figure S1, orange dots), suggesting the stability and reliability of the analytical procedures.

### 4.4. Metabolomics Analysis by RP-LC-QTOF-MS

Sample extraction was conducted by taking 50 µL of plasma sample, and 150 µL of methanol (at −20 °C) was added and vortexed at 3200 rpm for 3 min. The samples were incubated at −20 °C for 20 min and centrifuged at 13,000 rpm, 4 °C for 10 min. Metabolomic analysis was performed using an LC-QTOF-MS Agilent 6545 instrument, (Agilent Technologies Inc., Santa Clara, CA, USA). Chromatographic separation was performed using a C18 column (InfinityLab Poroshell 120 EC-C18 100 × 2.1 mm, 1.9 µm) at 40 °C and a gradient elution composed of 0.1% (*v*/*v*) formic acid in Milli-Q water (Phase A) and 0.1% (*v*/*v*) formic acid in acetonitrile (Phase B) with a constant flow of 0.4 mL/min. The elution gradient started with 5% Phase B, increasing to 95% B during the first 15 min, and was kept constant for 1 min. Finally, the percentage of Phase B was reduced to 5% from minutes 16 to 18, remaining at the initial conditions for 4 min for a final analysis time of 22 min. Mass spectrometry detection was performed in the positive ionization mode at a scan range of 50 to 1100 *m*/*z*. The source parameters were as follows: gas temperature, 250 °C; drying gas, 12 L/min; nebulizer, 52 psi; sheath gas temperature, 370 °C; sheath gas flow, 11 L/min; VCap, 3000 V; and nozzle voltage, 1000 V. Two reference masses were used for the mass correction: *m*/*z* 121.0509 (C_5_H_4_N_4_) and *m*/*z* 922.0098 (C_18_H_18_O_6_N_3_P_3_F_24_) [89].

### 4.5. Metabolomics Analysis by GC-QTOF-MS

For gas chromatography mass spectrometry analysis, 50 µL of the previous extract was dried under SpeedVac (Thermo Scientific, Waltham, MA, USA) for 1 h at 35 °C. Then, 20 µL of *O*-methoxyamine in pyridine (15 mg/mL) was added and vortexed at 3200 rpm for 5 min and left in the dark at room temperature for 16 h. For the silylation process, 20 µL of *N*,O-bis(trimethylsilyl)trifluoroacetamide (BSTFA) with 1% trimethylchlorosilane (TMS) was added, followed by agitation for 5 min and incubation at 70 °C for 1 h. Finally, 100 µL of methyl heptadecanoate-D33 was added as the internal standard (5 mg/L).

Data were acquired using an Agilent Technologies 7890 B GC system coupled with a time-of-flight mass spectrometer (Agilent Technologies 7250), (Agilent Technologies Inc., Santa Clara, CA, USA). A total of 1 µL of the derivatized sample was injected with a split ratio of 30 onto an Agilent Technologies HP-5MS column (30 m, 0.25 mm, 0.25 µm) at a constant flow of 0.7 mL/min. The oven temperature was 60 °C (1 min) at a rate of 10 °C/min up to 325 °C (10 min). The electron ionization source was set at 70 eV, and the acquisition was from 50 to 600 m/z at 5 spectra/s [89].

### 4.6. Quality Assurance (QA) and Quality Control Samples (QC)

A quality control sample was used for QA/QC purposes. QC was prepared from 10 µL of each sample. It was used to determine the reproducibility of sample preparation and stability of the analytical platform used. Subsequently, QC samples were analyzed every tenth of the randomly injected samples.

### 4.7. Data Processing and Analysis

The raw data obtained by LC-QTOF-MS were processed using the Agilent MassHunter Profinder software (version 10.0) to perform deconvolution, alignment, and integration. The GC-MS-QTOF data were processed using Agilent MassHunter Unknowns Analysis, Agilent Mass Profiler Professional, and Agilent MassHunter Quantitative B.10.0 software. These programs were used to deconvolute, align, integrate, and identify the samples’ metabolites.

The discriminating metabolites between the different biological stages and subtypes were defined using multivariate and univariate statistical analyses using the SIMCA 18, MetaboAnalyst (https://www.metaboanalyst.ca/ accessed on 5 February 2025) and MATLAB R2021b software (version 9.11.0.1769968), respectively. A Principal Component Analysis (PCA) was performed to visualize the trends between groups and verify the quality of the data. Similarly, partial least squares discriminant analysis (PLS-DA) was performed to obtain Variable Importance in Projection (VIP) values. Metabolites with a *p*-value, false discovery rate (FDR)-corrected *p*-value < 0.05, and/or VIP > 1 were considered significant and selected for evaluation as potential biomarkers using Receiver Operating Characteristic (ROC) curves. The analysis was performed using both classical univariate and multivariate analyses based on PLSDA.

### 4.8. Metabolite Identification

Identification of the significant features obtained by LC-QTOF-MS was performed based on the exact mass, molecular formula, mass spectra, and retention times. Each metabolite was reported with a confidence level according to the Metabolomics Standards Initiative (MSI). Where the highest level, Level 1, is assigned to metabolites identified by chemical standards; Level 2 for those with spectral coincidence in reference databases; Level 3 for putative compounds that have concordance in molecular formula and MS/MS data; and Level 4 for metabolites that match only in MS1 in online databases [90].

To confirm the metabolite annotation, the MS-DIAL 5.1.230912, LipidAnnotator version 10.0, and Sirius 5.8.3 software were used. The metabolites obtained by GC-MS-QTOF were identified using the “Fiehn GC-MS Metabolomics RTL Library” version 2011, which considers the coincidence in retention time, mass spectrum, and retention indices (RI) of fatty acid methyl esters (FAMES) or C7 to C20 alkanes.

### 4.9. Amino Acid Profiling

The samples were analyzed using an Agilent Technologies 1260 Liquid Chromatography system coupled to a 6470 triple quadrupole mass analyzer with electrospray ionization (Agilent Technologies Inc., Santa Clara, CA, USA). A total of 3 µL of the sample was injected onto an InfinityLab Poroshell 120 HILIC-Z, 2.1 x 100 mm, 1.9 µm column at 30 °C. The gradient elution was composed of Phase A, 10% (200 mM ammonium format in Milli-Q water, pH 3) with 90% water, and Phase B was 90% acetonitrile and 10% (200 mM ammonium format in water, pH 3) with a constant flow rate of 0.6 mL/min. The chromatographic elution gradient started with 100% Phase B and gradually decreased to 70% B by minute 10. Subsequently, the system returned to the initial conditions, with 100% Phase B, and was maintained in that state for an additional 5 min to ensure column stabilization. Mass spectrometry detection was performed in the Multiple Reaction Monitoring (MRM) mode at a capillary voltage of 3500 V, with an ESI source in positive mode. Nitrogen was used as the nebulizing gas at 45 psi with a gas temperature of 300 °C and a flow rate of 7 L/min. The sheath gas temperature was 400 °C at a flow rate of 11 L/min. Nitrogen (99.999% purity) was used as collision gas. The multiple reaction-monitoring transitions for each compound are listed in Supplementary Table S6.

Metabolites with an adjusted *p*-value for FDR (p-FDR) and/or an adjusted *p*-value for Bonferroni (*p*-Bonf) < 0.05, as well as those with a fold change greater than 1.0 or less than 0.9, were explored using Receiver Operating Characteristic (ROC) analysis. The analysis was performed using MetaboAnalyst (https://www.metaboanalyst.ca/ accessed on 15 April 2025).

## 5. Conclusions

This study provides comprehensive evidence of progressive metabolic reprogramming that occurs in BC, highlighting distinct alterations associated with both molecular subtypes and clinical stages. Through untargeted metabolomic profiling, we identified specific metabolic signatures, including disruptions in carbohydrate, lipid, and amino acid metabolism, that differentiate patients with BC from healthy individuals and evolve with disease progression. Notably, Luminal B tumors exhibit a hybrid metabolic phenotype characterized by enhanced glycolysis and lipid metabolism, which may help explain their more aggressive clinical behavior. In parallel, stratification by TNM stage revealed clear metabolic trends, such as phospholipid remodeling and the emergence of candidate diagnostic metabolites in early-stage disease. These findings support the potential of metabolomic approaches to contribute to early detection, disease monitoring, and molecular classification of BC.

## Figures and Tables

**Table 1:** Subset of breast cancer samples without thyroid disease.

				Biological Subtype				
Characteristics	Breast Cancer *n* = 63	Control Group *n* = 9	*p*	Luminal A *n* = 18	Luminal B *n* = 22	HER2 *n* = 13	Basal-Like *n* = 10	*p*
Age (M, SD)	62.1 ± 10.9	54.5 ± 12.9	0.06	57.4 ± 9.1	67.5 ± 9.7	62 ± 11.4	58.9 ± 11.8	0.019 *
BMI (M, SD)	25.8 ± 4.6	25.7 ± 3.1	0.91	25.6 ± 5	26.7 ± 5.3	25.5 ± 4	25 ± 2.9	0.551
T2DM (%)	3 (4.1)	0	0.5	0	1 (1.6)	2 (3.2)	0	0.206
Dyslipidemia (%)	14 (19.4)	2 (2.7)	1	5 (7.9)	5 (7.9)	3 (4.8)	1 (1.6)	0.758
Menopause	8 (11.1)	2 (2.7)	0.61	3 (4.8)	3 (4.8)	0	2 (3.2)	0.586
Postmenopause	46 (63.8)	4 (3.871)		11 (17.45)	18 (28.6)	11 (17.45)	6 (9.6)	
Premenopause	9 (12.5)	3 (4.1)		4 (6.3)	1 (1.6)	2 (3.1)	2 (3.1)	
Characteristics				TNM tumor stage				
				IA *n* = 10	IIA-IIB *n* = 22	IIIA-IIIC *n* = 18	IV *n* = 4	*p*
Age (M, SD)				65.05 ± 8.5	60.8 ± 10.4	65.2 ±12.2	76.2 ± 4.2	0.281
BMI (M, SD)				25.6 ± 5.3	25.8 ± 4.4	25.7 ± 4.7	30.7 ± 5.7	0.81
T2DM (%)				0	1 (1.6)	1 (1.6)	1 (1.6)	0.04 *
Dyslipidemia (%)				2 (3.1)	7 (11.1)	2 (3.1)	2 (3.2)	0.07
Hormonal status								
Menopause				1 (1.6)	3 (4.8)	1 (1.6)	0	0.492
Postmenopause				8 (12.7)	18 (28.5)	13 (20.6)	2 (3.2)	
Premenopause				0	6 (9.5)	2 (3.2)	0	

Abbreviations: M: mean; SD: standard deviation; BMI: body mass index; T2DM: type 2 diabetes mellitus; TNM: staging system that stands for Tumor, Node, and Metastasis. * Significant *p*-value < 0.05

## Data Availability

The data presented in this study are available at the NIH Common Fund’s National Metabolomics Data Repository (NMDR) website, the Metabolomics Workbench, https://www.metabolomicsworkbench.org (accessed on 15 April 2025), where they have been assigned Project ID PR002466 doi: 10.21228/M8W82G.

## Supplementary Materials

The following supporting information can be downloaded at https://www.mdpi.com/article/10.3390/ijms26157230/s1.
