# Supplementary material for: Metabolomic Analysis of Breast Cancer in Colombian Patients: Exploring Molecular Signatures in Different Subtypes and Stages
Source: Int J Mol Sci. 2025 Jul 26;26(15):7230. doi: 10.3390/ijms26157230 (PMC12346374; doi:10.3390/ijms26157230)
Supplement: Supplementary file 1 [file ijms-26-07230-s001.zip › Supplementary material.pdf]

Table S1. *Characteristics of the patients and tumors*

Characteristics	Breast Cancer n = 141	Control Group n = 14	p	Biological subtype Luminal A n = 41	Luminal B n = 69	HER2 n = 17	Basal Like n = 14	p
Age (M, SD)	65,1 ± 11,1	51,7 ± 13,7	<0.0009*	63,4±12,06	67,7 ± 9,5	60,3 ± 12,5	62,7 ± 11,7	0,071
BMI (M, SD)	26,7 ± 4,7	24,8 ± 3,1	0,4	26,5 ± 5,2	27,14 ± 4,6	25,4 ± 4,03	26,61 ± 4,3	0,551
DM T2 (%)	14 (9,032)	2 (1,29)	0,609	4 (2,83)	6 (4,25)	3 (2,13)	1 (0,7)	0,714
Thyroid disease	36 (23,2)	4 (2,5)	0,804	11 (7,8)	21 (14,9)	2 (1,42)	2 (1,42)	0,438
Dyslipidemia	36 (23,22)	2 (1,23)	0,85	11 (7,8)	16 (11,35)	5 (3,55)	4 (2,84)	0,932
Hormonal status								
Menopause	11 (7,097)	3 (1,935)		4 (2,84)	5 (3,55)	0	2 (1,42)	
Postmenopause	117 (75,484)	6 (3,871)	0,292	31 (21,99)	61 (43,26)	15 (10,64)	10 (7,1)	0,546
Premenopause	13 (8,387)	5 (3,226)		6 (4,25)	3 (2,13)	2 (1,41)	2 (1,41)	
**Characteristics**			**TNM tumor stage**					
			**In situ** *n* = 17	**IA** *n* = 23	**IIA-IIB** *n* = 64	**IIIA-IIIC** *n* = 31	**IV** *n* = 6	***p***
Age (M, SD)			60,66 ± 9,9	67,3 ± 9,1	65,5 ± 67,1	65,2 ±13,5	69,3 ±14,8	0,235
BMI (M, SD)			26,7 ± 5,2	26,05 ±4,2	26,9 ± 4,8	26,3 ± 4,9	29,1 ± 3,4	0, 635
TII DM			2 (1,42)	1 (0,71)	8 (5,67)	2 (1,42)	1 (0,71)	0,727
Hormonal status								
Thyroid disease			5 (3,55)	6 (4,25)	18 (12,76)	5 (3,55)	2 (1,42)	0,804
Menopause			3 (2,13)	2 (1,42)	5 (3,55)	1 (0,71)	0	0,516
Postmenopause			12 (8,51)	21 (14,9)	52 (36,88)	26 (18,44)	6 (4,25)	0, 635
Premenopause			2 (1,4)	0	7 (4,97)	4 (2,84)	0	0,727

M: mean; SD: Standard Deviation; BMI: Body Mass Index; DM T2: Type 2 Diabetes mellitus; TNM: staging system that stands for Tumor, Node and Metastasis

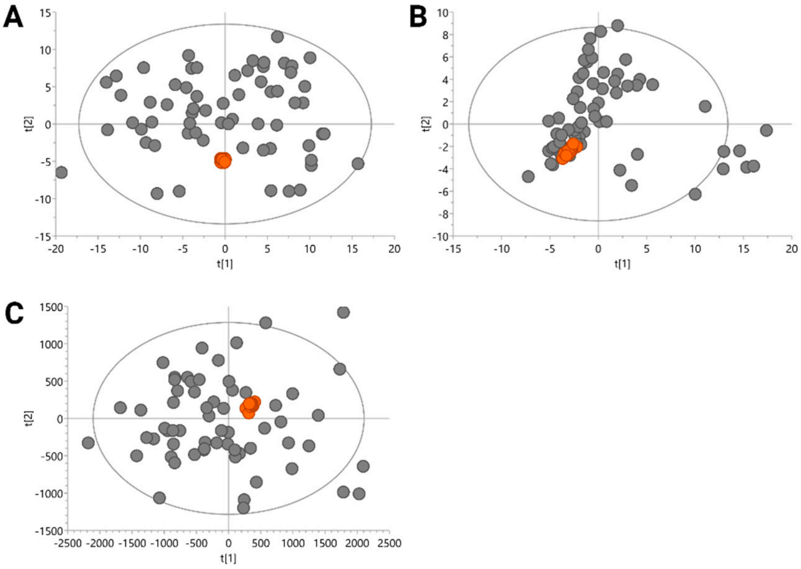

Figure S1. PCA models for metabolic analysis A) LC-QTOF-MS (+): R^2: 0.715, Q^2: 0.131, (B) GC-QTOF-MS: R^2: 0.573, Q^2: 0.379; (C) Amino acid profile: R^2: 0.909, Q^2: 0.752. Dots in orange denote quality control, gray dots correspond to samples.

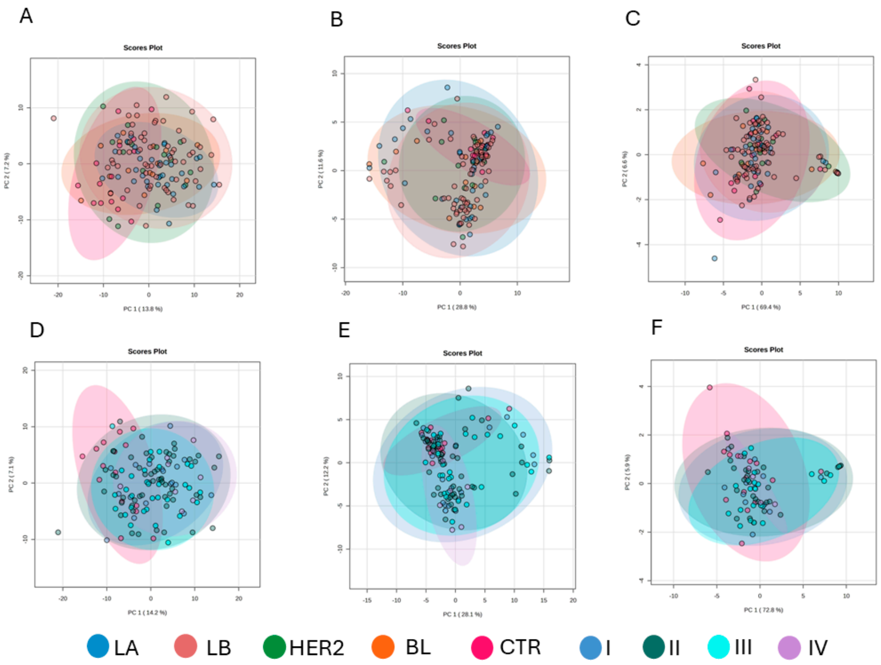

Figure S2. PCA models for metabolic analysis between subtypes and stages: A) LC-QTOF-MS (+): R^2: 0.715, Q^2: 0.131, (B) GC-QTOF-MS: R^2: 0.573, Q^2: 0.379; (C) Amino acid profile: R^2: 0.909, Q^2: 0.752. D) LC-QTOF-MS (+): R^2: 0.582, Q^2: 0.165, E) GC-QTOF-MS: R^2: 0.582, Q^2: 0.373; F) Amino acid profile: R^2: 0.670, Q^2: 0.346
