# Supplementary material for: Metabolomic Analysis of Breast Cancer in Colombian Patients: Exploring Molecular Signatures in Different Subtypes and Stages
Source: Int J Mol Sci. 2025 Jul 26;26(15):7230. doi: 10.3390/ijms26157230 (PMC12346374; doi:10.3390/ijms26157230)
Supplement: Supplementary file 1 [file ijms-26-07230-s001.zip › Table S6. Multiple Reaction Monitoring transitions for amino acid analysis..pdf]

Table S6. Multiple Reaction Monitoring transitions for amino acid analysis.

**Amino acid**	**Transition (*m*/*z*)**	**RT (min)**	**Fragmentor**	**CE (V)**
Trp	205.1 -> 146	3.71	25	15
Arg	175.1 -> 70.1	8.27	75	28
Phe	166.1 -> 120.1	3.51	25	5
His	156.1 -> 110	7.44	25	12
Met	150.1 -> 56.1	4.38	22	9
Glu	148.1 -> 84.1	7.04	75	16
Lys	147.1 -> 84	8.71	50	16
Ile	132.1 -> 86.1	4.10	25	8
Leu	132.1 -> 86.1	3.81	25	8
Hyp	132.1 -> 86	5.65	79	27
Cr	132.1 -> 44	5.56	28	10
Thr	120.1 -> 56.2	5.71	25	10
Val	118.1 -> 72.1	4.97	25	8
Pro	116.1 -> 70.1	5.03	50	16
Ser	106 -> 60.1	6.21	25	8
Ala	90.1 -> 44.1	5.63	25	8
Sar	90.1 -> 44	5.48	44	19
Gly	76 -> 30.1	6.03	25	5
