# Supplementary figures and images for: Metabolomic Analysis of Breast Cancer in Colombian Patients: Exploring Molecular Signatures in Different Subtypes and Stages
Source: Int J Mol Sci. 2025 Jul 26;26(15):7230. doi: 10.3390/ijms26157230 (PMC12346374; doi:10.3390/ijms26157230)

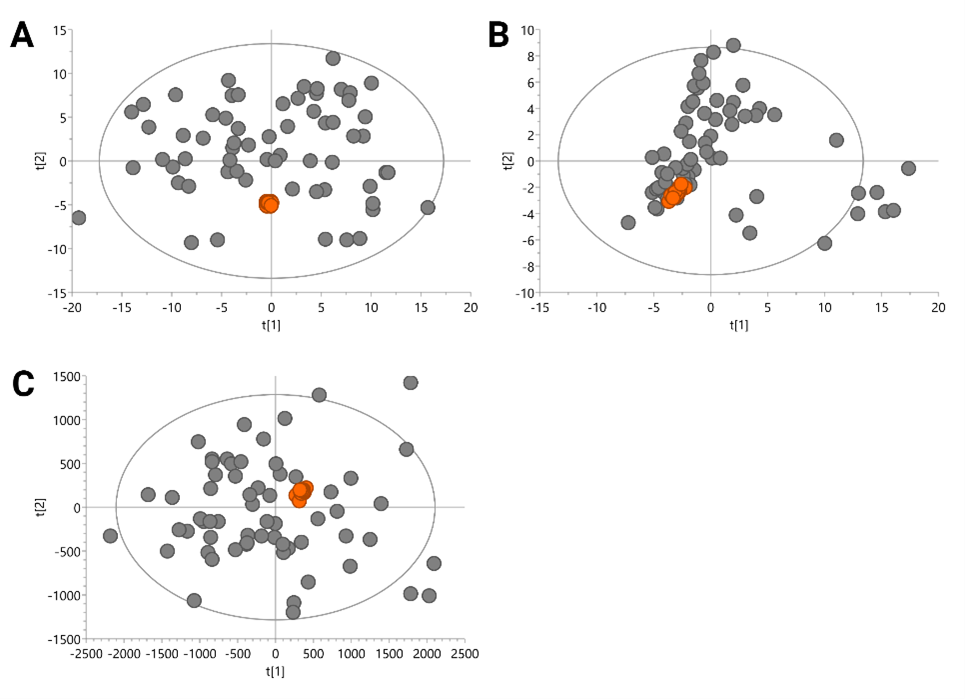

Supplement: Supplementary file 1 [file ijms-26-07230-s001.zip › Figure S1. PCA models for metabolic analysis .tiff]

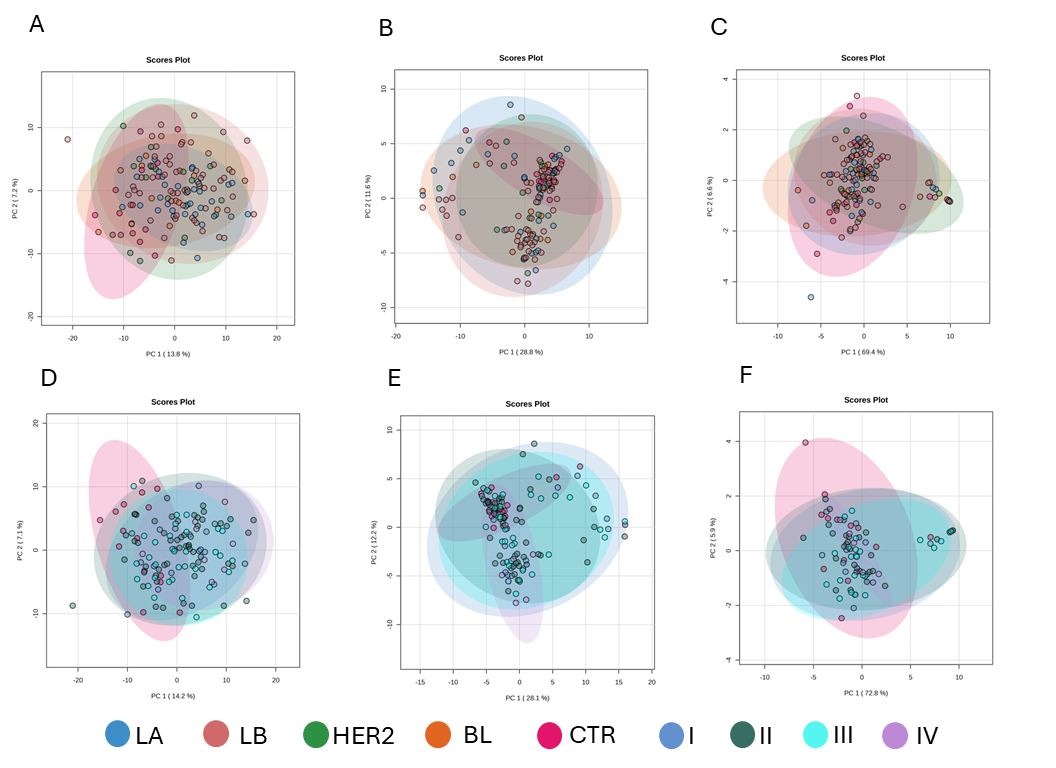

Supplement: Supplementary file 1 [file ijms-26-07230-s001.zip › Figure S2_PCA samples.png]
